# The Association Between the Frequency of Agriculture and Control of Chronic Diseases Among Regular Patients in Rural Community Hospitals: A Cross-Sectional Study

**DOI:** 10.7759/cureus.62849

**Published:** 2024-06-21

**Authors:** Ryuichi Ohta, Toshihiro Yakabe, Hiroshi Adachi, Chiaki Sano

**Affiliations:** 1 Communiy Care, Unnan City Hospital, Unnan, JPN; 2 Family Medicine, Unnan City Hospital, Unnan, JPN; 3 Community Medicine Management, Faculty of Medicine, Shimane University, Izumo, JPN

**Keywords:** rural health, hypertension, agriculture, exercise, aged, chronic disease

## Abstract

Introduction

The importance of exercise in chronic disease management among older people is paramount, especially as the global population ages and the prevalence of chronic diseases such as cardiovascular disease, diabetes, and arthritis increases. Regular physical activity enhances cardiovascular health, improves metabolic function, and alleviates symptoms of musculoskeletal disorders. As a form of exercise, agriculture provides physical and mental benefits for older adults. However, its impact on chronic disease management can be mixed, as the physical demands and potential stress associated with agricultural tasks can exacerbate certain health conditions.

Method

This cross-sectional study was conducted in Unnan City, a rural area in Japan, focusing on residents aged 40 and older who regularly visited Unnan City Hospital. Data were collected from 647 participants between September 1, 2023, and November 31, 2023, through questionnaires and electronic medical records. The primary outcome was the management of chronic diseases, assessed through hypertension, dyslipidemia, diabetes mellitus, and obesity control. Participants reported their frequency of agricultural activities, exercise, eating habits, and sleep. Statistical analyses included t-tests, Mann-Whitney U tests, and multivariate logistic regression models.

Results

Participants engaging frequently in agricultural activities were younger, had better hypertension control, and reported healthier eating habits and sleep patterns. Frequent agricultural activities were associated with a lower likelihood of hypertension (OR: 0.62, 95% CI: 0.39-0.97, p = 0.034). Older age (≥75 years), higher BMI (≥25), and a Charlson comorbidity index (CCI) score of ≥5 were significantly associated with hypertension. No significant associations were found between hypertension and other health-related variables such as healthy eating, adequate sleep, and regular exercise.

Conclusion

The study highlights the potential benefits of agricultural activities in managing chronic diseases, particularly hypertension, among older adults. However, the physical demands and possible social isolation associated with agricultural tasks require a nuanced approach to promoting these activities. Tailored, community-based agricultural programs that foster social interaction and support can enhance physical and mental health benefits. Future research should focus on longitudinal studies to confirm these findings and explore the long-term health outcomes of agricultural activities in diverse contexts.

## Introduction

The importance of exercise in chronic disease management among older people cannot be overstated [1]. As the global population ages, the prevalence of chronic diseases such as cardiovascular disease, diabetes, and arthritis increases [2]. Exercise has consistently been critical in preventing and managing these conditions [2]. Regular physical activity can enhance cardiovascular health, improve metabolic function, and alleviate symptoms of musculoskeletal disorders [3]. For older adults, maintaining an active lifestyle is crucial for preserving functional independence and quality of life [4]. Among the various forms of exercise, agriculture stands out as a unique and beneficial activity for older people, providing both physical exertion and mental engagement [5].

One of the exercises for older people is agriculture, which can be pursued for joy or as a means of livelihood [6]. Engaging in agricultural activities can encompass a range of tasks, from planting and harvesting crops to tending to livestock [7]. These activities require varying levels of physical effort, thus providing a practical way for older adults to stay active. Moreover, agriculture can offer psychological benefits by fostering a sense of purpose and connection to nature [8]. For many older adults, especially those in rural areas, agriculture is not just a hobby but an integral part of their daily lives and identity.

However, agriculture can have mixed effects on managing health conditions among older adults. On the one hand, the physical activity involved in agricultural work can contribute positively to managing chronic diseases by promoting cardiovascular fitness, muscle strength, and flexibility [9]. On the other hand, the physical demands and potential stress associated with agricultural tasks can exacerbate certain health conditions, mainly if the work is strenuous or performed in challenging conditions [9]. Additionally, the impact of agricultural activities on mental health can vary, influenced by factors such as social support, financial stress, and the individual's overall health status [10].

The previous study in rural contexts shows that doing agriculture frequently can be associated with high loneliness, impinging on people’s health [10]. This finding highlights a paradox where the solitary nature of some agricultural tasks can lead to social isolation, negatively affecting mental health and potentially offsetting the physical health benefits. Loneliness has been linked to a range of adverse health outcomes, including increased risk of cardiovascular disease, depression, and cognitive decline [11-13]. Therefore, while agriculture can benefit physical health, its impact on mental health must be carefully considered.

Conversely, other studies show the better effects of agriculture on people’s health conditions. Research has indicated that agricultural activities can reduce symptoms of depression and anxiety, improve overall mood, and enhance social connectedness when performed in a communal setting [14]. Community gardens, for instance, provide opportunities for social interaction, collaboration, and mutual support among participants [14]. These positive social experiences can mitigate feelings of loneliness and contribute to better mental health outcomes, demonstrating the potential of agriculture to support holistic health.

This study focuses on the prevalence of chronic diseases and investigates the association between agricultural activities and the prevalence of common chronic diseases. By examining both the physical and mental health impacts of agricultural engagement, this research aims to provide a nuanced understanding of how such activities can be optimized to benefit older adults. The findings will offer valuable insights for healthcare providers, policymakers, and community planners seeking to promote healthy aging and improve the quality of life for older populations through tailored, context-specific interventions in agriculture and other forms of exercise.

## Materials and methods

Method

This cross-sectional study was performed with rural citizens who regularly visited a rural Japanese community hospital to clarify the association between the frequency of agriculture and the control of chronic diseases among regular patients in rural community hospitals.

Setting

Unnan City, a rural municipality in southeastern Shimane Prefecture, Japan, had a population of 37,637 in 2020, comprising 18,145 men and 19,492 women. Notably, 39% of the population was aged over 65, and this demographic is projected to increase to 50% by 2025. The city is served by 16 clinics, 12 home care stations, three visiting nurse stations, and Unnan City Hospital, a public facility with 281 beds, including 160 acute care beds, 43 comprehensive care beds, 30 rehabilitation beds, and 48 chronic care beds [15]. Care managers in Unnan City operate independently or as part of home care stations, providing a vital link between home care patients, their families, and other healthcare professionals. They assess and coordinate the necessary care services for their patients. Home care workers, based at home care stations, assist patients with physical care, daily living activities, and transportation services [16].

Participants

All patients over 40 years of age who were regular visitors to the Department of General Medicine at Unnan City Hospital between September 1, 2023, and November 31, 2023, were included in the study [12]. Data collection involved extracting information from the electronic medical records of patients who visited the hospital regularly for chronic disease management or annual health checks. The study included patients aged 40 and above who consistently visited the Department of General Medicine for chronic conditions such as hypertension, dyslipidemia, diabetes mellitus, or obesity or for their annual health checks and gave informed consent. Patients were excluded if they had incomplete or missing data in their electronic medical records, did not regularly visit the Department of General Medicine, were under 40 years old, did not provide informed consent, or visited the hospital for acute conditions rather than chronic disease management or annual health checks. This ensured the study focused on a relevant and well-defined patient population.

Data collection

Primary Outcome

The primary outcome was chronic disease conditions such as hypertension, dyslipidemia, diabetes mellitus, and obesity. The definition of diagnoses of chronic diseases was set for each condition. Regarding hypertension, the diagnosis is set as more than 130/80 at the outpatient department. Regarding dyslipidemia, the diagnosis is set as low-density lipoprotein (LDL) cholesterol of more than 140 mg/dL at the outpatient department. Regarding diabetes mellitus, the diagnosis is set as hemoglobin A1C, which is more than 6.5% in the outpatient department.

Independent Variables

Regarding agricultural activities, participants were asked about the frequency of agricultural activities in their community. The question was “How often do you engage in agricultural activities in your daily life?". (Here, "agricultural activities" include not only the farming done by farmers as an economic activity but also the gardening or cultivation of crops at home or in community gardens by non-farmers as a hobby or leisure activity.) The participants answered the question with not one time or less weekly, two to three times weekly, four to five times weekly, and more than five times weekly [12,17].

Regarding exercise, the participants were asked as follows: “Do you engage in regular exercise?" (For this question, "exercise" refers to physical activity performed at least three times a week, sustained for a minimum of ten minutes per session.) The participants answered the question with a yes or no.

Regarding eating habits, the participants were asked as follows: “How many days per week do you have at least two meals consisting of three components: a staple food (such as rice, bread, or noodles), a main dish (using meat, fish, eggs, or soy products), and a side dish (prepared with vegetables, mushrooms, tubers, or seaweed)?” The participants answered the question with not one time or less weekly, two to three times weekly, four to five times weekly, and more than five times weekly [12,17].

Regarding sleep, the participants were asked as follows: “Do you make an effort to get sufficient sleep?" (In this context, "sufficient sleep" is defined as a minimum of six hours per night.) The participants answered the question with a yes or no.

Background information on the participants was gathered from the electronic patient records at Unnan City Hospital [12]. Collected data included the patient's age, sex, body mass index for nutritional assessment, serum creatinine level (mg/dL), estimated glomerular filtration rate (eGFR) (mL/min/1.73 m^2^) for evaluating renal function, and Charlson comorbidity index (CCI) for assessing the severity of comorbidities such as heart failure, myocardial infarction, asthma, chronic obstructive pulmonary disease, kidney disease, liver disease, diabetes mellitus, brain infarction, brain hemorrhage, hemiplegia, connective tissue diseases, dementia, and cancer [18]. The laboratory data were obtained from the participants' most recent visits for chronic disease management or annual health checks [12].

Statistical analysis

Student’s t-test was employed for analyzing parametric data, while the Mann-Whitney U test was used for nonparametric data. Various variables were categorized as follows: agricultural activities (infrequent: once or less weekly, two to three times weekly; frequent: four to five times weekly, more than five times weekly), age (75 years and older or younger than 75 years), eating habits (less frequent: once or less weekly, two to three times weekly; frequent: four to five times weekly, more than five times weekly), CCI (greater than five or five and below), and BMI (25 and over, or less than 25). A univariate regression model was utilized to determine the association between hypertension and the independent variables. To further explore the relationship between agricultural activities and chronic diseases such as hypertension, diabetes, and dyslipidemia, a multivariate logistic regression analysis was conducted. This model included only those chronic diseases and health-related variables significantly correlated with agricultural activities in the univariate regression analysis (p-value < 0.1) and had been considered in previous studies. Participants with missing data were excluded from the analysis. Statistical significance was defined as p < 0.05. All statistical analyses were performed using EZR (Saitama Medical Center, Jichi Medical University, Saitama, Japan), a graphical user interface for R (The R Foundation, Vienna, Austria) [19].

Ethical considerations

The hospital ensured the anonymity and confidentiality of patient information utilized in this study. Information about the study was made available on the hospital's website without revealing any patient details. Contact information for a hospital representative was also provided on the website to address any inquiries about the study. All participants were informed about the study's purpose and gave their informed consent. The study protocol was approved by the Unnan City Hospital Clinical Ethics Committee (approval code: 20230010).

## Results

Participant selection

Between September 1, 2023, and November 31, 2023, the general medicine department regularly followed 1,024 patients. The questionnaires were sent to all the patients. In total, 647 participants who answered the questionnaires were included in this study [12].

Demographics of the participants

The participants were divided into two groups based on the frequency of their agricultural activities: not frequent (engaging in agricultural activities one time or less weekly, or two to three times weekly) and frequent (four to five times weekly, or more than five times weekly). Significant differences were observed between these groups in various demographic factors: The mean age of the participants was 71.26 years (SD: 12.18), with those engaged in frequent agricultural activities being younger on average (mean age: 69.53 years) compared to those in the not frequent group (mean age: 77.17 years) (p < 0.001). A more significant proportion of participants aged 75 years and over were in the not frequent agriculture group (61.0%) compared to the frequent agriculture group (37.5%) (p < 0.001). A higher percentage of male participants was found in the not frequent group (58.9%) compared to the frequent group (42.6%) (p = 0.001). The mean BMI was 23.00 (SD: 3.81), with no significant difference between the two groups (p = 0.449). The groups had no significant differences in hemoglobin levels, hemoglobin A1c, LDL cholesterol, or albumin. Participants who engaged in frequent healthy eating were significantly more likely to engage in frequent agricultural activities (23.8%) compared to those who did not (15.8%) (p = 0.041). A more significant proportion of participants in the frequent agriculture group reported adequate sleep (81.0%) compared to those in the not frequent group (90.4%) (p = 0.008). Hypertension was more prevalent in the not frequent agriculture group (76.0%) compared to the frequent group (63.3%) (p = 0.004). There were no significant differences in the prevalence of dyslipidemia or diabetes mellitus between the two groups. The CCI indicated that participants with a CCI ≥ 5 were more common in the not frequent group (43.2%) compared to the frequent group (30.9%) (p = 0.007) (Table 1).

**Table 1:** The demographics of the participants were categorized based on the amount of loneliness BMI, body mass index; Charlson Comorbidity Index, CCI; eGFR, estimated glomerular filtration rate; SD, standard deviation; HTN, hypertension; DL, dyslipidemia; DM, diabetes mellitus; LDL, low density lipoprotein

Factor	Total	Not frequent agriculture	Frequent agriculture	p-value
N	647	146	501	
Age, mean (SD)	71.26 (12.18)	77.17 (6.97)	69.53 (12.82)	<0.001
Age 75 and over	277 (42.8)	89 (61.0)	188 (37.5)	<0.001
Male sex (%)	299 (46.3)	86 (58.9)	213 (42.6)	0.001
BMI, mean (SD)	23.00 (3.81)	22.79 (3.08)	23.06 (4.00)	0.449
BMI 25 and over	181 (28.0)	37 (25.3)	144 (28.7)	0.464
Hemoglobin, g/L	13.28 (1.44)	13.27 (1.40)	13.28 (1.45)	0.937
Hemoglobin A1c	5.77 (0.56)	5.80 (0.57)	5.76 (0.55)	0.522
LDL	106.26 (23.85)	104.47 (21.16)	106.78 (24.57)	0.304
Albumin	4.10 (0.41)	4.03 (0.36)	4.11 (0.42)	0.034
Healthy eating (%)				
Less than 1 time weekly	361 (55.8)	99 (67.8)	262 (52.3)	<0.001
2 to 3 times weekly	144 (22.3)	24 (16.4)	120 (24.0)	
4 to 5 times weekly	116 (17.9)	23 (15.8)	93 (18.6)	
Every day	26 (4.0)	0 (0.0)	26 (5.2)	
Frequent Healthy eating (%)	142 (21.9)	23 (15.8)	119 (23.8)	0.041
Exercise (%)	267 (41.3)	70 (47.9)	197 (39.3)	0.07
Good sleep (%)	538 (83.2)	132 (90.4)	406 (81.0)	0.008
Hypertension (%)	428 (66.2)	111 (76.0)	317 (63.3)	0.004
Dyslipidemia (%)	388 (60.0)	88 (60.3)	300 (59.9)	1
DM (%)	130 (20.1)	34 (23.3)	96 (19.2)	0.292
CCI ≥ 5 (%)	218 (33.7)	63 (43.2)	155 (30.9)	0.007
CCI (%)				
0	20 (3.1)	0 (0.0)	20 (4.0)	
1	57 (8.8)	0 (0.0)	57 (11.4)	
2	82 (12.7)	11 (7.5)	71 (14.2)	
3	142 (21.9)	38 (26.0)	104 (20.8)	
4	128 (19.8)	34 (23.3)	94 (18.8)	
5	107 (16.5)	30 (20.5)	77 (15.4)	
6	64 (9.9)	21 (14.4)	43 (8.6)	
7	34 (5.3)	11 (7.5)	23 (4.6)	
8	10 (1.5)	1 (0.7)	9 (1.8)	
9	3 (0.5)	0 (0.0)	3 (0.6)	

The result of the logistic regression analysis

The univariate analysis showed that only hypertension was significantly associated with frequent agricultural activities. The multivariate logistic regression analysis focused on significant associations between hypertension, agricultural activities, and other healthy behavior-related variables. Participants engaging in frequent agricultural activities were less likely to have hypertension (OR: 0.62, 95% CI: 0.39-0.97, p = 0.034). Participants aged 75 and over were more likely to have hypertension (OR: 1.63, 95% CI: 1.07-2.49, p = 0.024). A BMI of 25 or higher was significantly associated with hypertension (OR: 2.09, 95% CI: 1.38-3.17, p = 0.00046). A CCI score of 5 or higher was also significantly associated with hypertension (OR: 1.88, 95% CI: 1.21-2.93, p = 0.0051). No significant associations were found between hypertension and healthy eating, adequate sleep, regular exercise, or albumin levels (Table 2).

**Table 2:** The multivariate logistic regression model with hypertension, agricultural activities, and other health-related factors BMI, body mass index; CCI, Charlson Comorbidity Index; CI, confidential interval

Factor	Odds ratio	95% CI	P value
Frequent agricultural activities	0.62	0.39-0.97	0.034
age≧75	1.63	1.07-2.49	0.024
BMI≧25	2.09	1.38-3.17	0.00046
CCI≧5	1.88	1.21-2.93	0.0051
Healthy eating	1.47	0.95-2.28	0.084
Adequate sleep	0.82	0.52-1.32	0.42
Regular exercise	1.20	0.84-1.71	0.32
Albumin	1.13	0.73-1.74	0.58

## Discussion

The findings of this study highlight the multifaceted relationship between agricultural activities and managing chronic diseases among older adults. Engaging in frequent agricultural activities was significantly associated with better hypertension control, aligning with previous research that underscores the cardiovascular benefits of regular physical activity [20]. The physical exertion involved in agriculture likely contributes to improved cardiovascular fitness and reduced blood pressure, as studies on exercise and cardiovascular health suggest [21]. In the rural context, agriculture is one of the vital activities for the sustainability of communities [22]. Considering the possibility of the effectiveness of agriculture, rural communities should drive agricultural activities to prevent hypertension.

Interestingly, this study found that frequent agricultural activities were more common among younger participants within the study group, which could be attributed to the demanding nature of agricultural work. This age-related difference in activity levels underscores the need to tailor health interventions to accommodate the physical capabilities of older adults [23]. The finding that participants aged 75 and over were more likely to have hypertension aligns with existing literature on the age-related increase in hypertension prevalence [24]. In addition, older people tend to feel lonely and isolated in rural communities, based on the previous article [10]. Promoting agricultural activities’ effect on hypertension should be nuanced and adjusted according to generation differences in interactions with communities and their perceptions.

The role of BMI in hypertension management is also significant. Participants with a BMI of 25 or higher were more likely to have hypertension, reinforcing the well-documented relationship between obesity and hypertension [25]. In Japan, compared to Western countries, there are fewer obese patients, and a higher BMI is assessed at the cutoff point of 25 [26]. In this research, the cutoff shows a significant association with hypertension, which can mean the difference in the assessment of BMI among countries [26]. On the other hand, higher BMI can be associated with less loneliness in rural context research [11]. Cultural issues can affect this association in Asia, such as that skinny figures are not healthy among middle to older generations [27]. Such consideration impinges on their interaction with others. The management of BMI, including health and social issues, is complicated, and each person’s perception of body shape is respected.

In this research, higher CCI and older age were associated with an increased likelihood of hypertension, highlighting the impact of multiple chronic conditions on health outcomes [18,28]. Multimorbidity can be critical, especially among older patients. In aging countries, multimorbidity and polypharmacy impinge on older people’s health [29]. Hypertension is one of the critical factors inducing multimorbidity. To promote older people’s health, multimorbidity should be prevented through regular exercise, including agricultural activities and increasing social interactions.

This study also provides insights into the mental health implications of agricultural activities. While the physical benefits are evident, the psychological impact is complex [6,9]. Previous studies have shown mixed results regarding the mental health effects of agriculture [7,30]. On the one hand, agricultural activities can reduce symptoms of depression and anxiety when performed in communal settings [14]. On the other hand, the solitary nature of some agricultural tasks can lead to social isolation and loneliness, particularly in rural contexts where social interaction may be limited [10]. The higher prevalence of loneliness among those engaged in less frequent agricultural activities suggests that social support and community engagement are crucial for maximizing the mental health benefits of agriculture [10].

Several limitations must be acknowledged in this study. Firstly, the cross-sectional design limits the ability to establish causality between agricultural activities and chronic disease management. Longitudinal studies are needed to confirm these associations and understand the long-term effects of agricultural engagement. Secondly, the study relied on self-reported data for agricultural activity frequency and health behaviors, which may be subject to recall and social desirability biases. Objective measures of physical activity and more comprehensive assessments of dietary habits and sleep quality could enhance the reliability of the findings. Thirdly, the study was conducted in a specific rural Japanese context, which may limit the generalizability of the results to other populations and settings with different agricultural practices and healthcare systems.

## Conclusions

This study contributes to the growing body of evidence supporting the benefits of agricultural activities for managing chronic diseases among older adults. Frequent engagement in agriculture was associated with better hypertension control, highlighting the potential of incorporating agricultural activities into health promotion strategies for older populations. However, the mixed effects on mental health and the significant influence of age, BMI, and comorbidities underscore the need for a holistic and individualized approach to health interventions. Healthcare providers and policymakers should consider agricultural work's physical and psychological demands and promote community-based agricultural programs that foster social interaction and support. Future research should explore the long-term health outcomes of agricultural activities and investigate strategies to mitigate the negative mental health impacts. Additionally, studies in diverse geographical and cultural contexts are needed to confirm the generalizability of these findings and inform tailored interventions. By addressing these gaps, we can better harness the potential of agriculture to support healthy aging and improve the quality of life for older adults globally.
